# Age Discrimination and Perceived Work Ability: The Mediating Effect of Self-Efficacy

**DOI:** 10.1093/geroni/igab046.3662

**Published:** 2021-12-17

**Authors:** Hyun Kang, Hansol Kim

**Affiliations:** 1 George Mason University, Fairfax, Virginia, United States; 2 National Rehabilitation Research & Training Center on Family Support, Pittsburgh, Pennsylvania, United States

## Abstract

As the number of older workers in the U.S. workforce increases, perceived work ability, which indicates a worker’s capacity to perform job-related tasks or to remain employed, becomes increasingly important. However, age discrimination may undermine the ability of older adults to remain active in the workplace as it poses a significant barrier to their work ability. The purpose of this study was to examine how age discrimination affects perceived work ability among older workers. We also evaluated the role of self-efficacy as a potential mediator between age discrimination and perceived work ability. Self-efficacy can contribute to older adults’ productive aging since it helps them view age-related situations more positively. Using 2,011 respondents (aged 50+) data from the 2018 Health and Retirement Study, structural equation modeling analysis was conducted. Our findings indicated that age discrimination had a direct negative effect on perceived work ability (B = -.230, p < .001). Older workers who experienced more age discrimination were more likely to have low levels of perceived work ability. The indirect effect of self-efficacy (B = -.177; 95% CI = -.240, -.135) was significant. Older workers who experienced more age discrimination were more likely to have low levels of self-efficacy, and this relationship led to lower levels of perceived work ability. These results suggest that greater efforts are required to reduce age discrimination and its negative consequence on perceived work ability and self-efficacy among older workers. Furthermore, age discrimination laws should be more explicitly enforced in the policy direction for older workers.

